# Induction of Tumor Necrosis Factor (TNF) Release from Subtypes of T Cells by Agonists of Proteinase Activated Receptors

**DOI:** 10.1155/2013/165453

**Published:** 2013-12-16

**Authors:** Haiwei Yang, Tao Li, Jifu Wei, Huiyun Zhang, Shaoheng He

**Affiliations:** ^1^Department of Urology, The First Affiliated Hospital of Nanjing Medical University, Nanjing, 300 Guangzhou Road, Jiangsu 210029, China; ^2^Department of Infectious Diseases, The First Affiliated Hospital of Shantou University Medical College, Shantou 515041, China; ^3^Allergy and Clinical Immunology Research Centre, The First Affiliated Hospital of Liaoning Medical University, Jinzhou, Liaoning 121001, China

## Abstract

Serine proteinases have been recognized as playing an important role in inflammation via proteinase activated receptors (PARs). However, little is known about the influence of serine proteinases and PARs on TNF secretion from highly purified T cells. We challenged T cells from human peripheral blood with serine proteinases and agonist peptides of PARs and measured the levels of TNF in culture supernatants by ELISA. The results showed that thrombin and trypsin, but not tryptase, stimulated approximately up to 2.5-fold increase in TNF release from T cells following 16 h incubation. Proteinase inhibitors and PAR-1 antagonist SCH 79797 almost completely abolished thrombin- and trypsin-induced TNF release from T cells. Agonist peptides of PAR-1, but not PAR-2 induced TNF release from T cells. Moreover, trypsin- and thrombin-induced upregulated expression of TNF was observed in CD4+, IL-4+, or CD25+ T cells, but not in IFN+ or IL-17+ T cells. The signaling pathways MAPK/ERK and PI3K/Akt are involved in the thrombin- and trypsin-induced TNF release from T cells. In conclusion, thrombin and trypsin can induce TNF release from IL-4+ and CD25+ T cells through activation of PAR-1 and therefore contribute to regulation of immune response and inflammation of the body.

## 1. Introduction

Proteinase-activated receptors (PARs) belong to a family of G-protein-coupled receptors with seven transmembrane domains activated via proteolytic cleavage by serine proteinases [1]. A total of four PARs have been identified and cloned. Among them, PAR-1 [2, 3], PAR-3 [4], and PAR-4 [5] are targets for thrombin, trypsin, and cathepsin G, whereas PAR-2 is resistant to thrombin but can be activated by trypsin, mast cell tryptase [6, 7], neutrophil elastase [8], and insect-derived proteinase [9].

PARs are expressed by various cells involved in inflammatory and immunological responses, such as vascular endothelial cells, epithelial cells, mast cells, T cells, monocyte, eosinophils, and neutrophils [10, 11]. In these cells, activation of PARs affects their main functions such as proliferation, degranulation, and release of inflammatory mediators [10, 11]. In our previous study [12], we have showed the expression of PAR-1, PAR-2, and PAR-3 on T cells, and thrombin-, trypsin-, and tryptase-induced interleukin (IL-6) release from T cells. It has also been reported that cytoplasmic free calcium and phospholipase C and protein kinase C activation are increased in T-leukemic cell lines following stimulation with thrombin or the thrombin receptor agonist peptide [13]. Thrombin and thrombin receptor agonist also enhanced CD69 expression and IL-2 productions by cross-linking T cell receptors in both Jurkat T cells and peripheral blood lymphocytes [14]. We, therefore, anticipated that thrombin, trypsin, and tryptase might induce TNF release from T cells through PARs.

TNF is a major proinflammatory cytokine that is thought to be important in the pathogenesis of asthma [15], food allergy [16], ocular allergy [17], and atopic dermatitis [18]. It has been reported that the increased number of TNF expressing cells and levels of TNF is observed in the bronchoalveolar lavage (BAL) and in the airways of asthmatics [19]. Inhaled TNF increases airway responsiveness to methacholine in asthmatic subjects associated with a sputum neutrophilia [20]. Since PARs, TNF, and T cells all play roles in inflammation, we believe, there must be some linkages between them. The aim of the present study is to investigate roles of thrombin, tryptase, trypsin, elastase, and agonist peptides of PARs in the secretion of TNF from purified human T cells and subtypes of T cells.

## 2. Materials and Methods

### 2.1. Reagents

Human thrombin, trypsin (specific activity: *∼*10,000 BAEE U/mg protein), soybean trypsin inhibitor (SBTI), and bovine serum albumin (BSA, fraction V) were purchased from Sigma (St Louis, MO, USA). Recombinant hirudin and human neutrophil elastase (specific activity: 20 MeO-Suc-Ala-Ala-Pro-Val-pNA U/mg protein) were obtained from Calbiochem (San Diego, CA, USA). Recombinant human Lung *β* tryptase (specific activity: *∼*1,000 N*α* CBZ-L-Lysine Thiobenzyl Ester U/mg protein) was from Promega (Madison, WI, USA). SCH 79797 was from Tocris Cookson (Ellisville, Mo, USA). Agonist peptides of PARs, and their reverse forms, and PAR-2 antagonist peptide FSLLRY-NH_2_ were synthesized in CL Bio-Scientific Inc. (Xi An, China). The sequences of the active and reverse peptides were PAR-1, SFLLR-NH_2_ and RLLFS-NH_2_, TFLLRN-NH_2_ and NRLLFT-NH_2_; PAR-2, SLIGKV-NH_2_ and VKGILS-NH_2_ as well as trans cinnamoyl (tc)-LIGRLO-NH_2_ and tc-OLRGIL-NH_2_; PAR-3, TFRGAP-NH_2_ and PAGRFT-NH_2_. RPMI 1640 and newborn calf serum (NCS) were obtained from GIBCO (Carlsbad, CA, USA). Ficoll-Paque Plus was from Amersham Biosciences (Uppsala, Sweden). PE-conjugated mouse anti-human CD3 monoclonal antibody, PE-conjugated goat-anti rabbit IgG, and TNF OptEIA ELISA kits were purchased from BD PharMingen (San Jose, CA, USA). TRIzol reagent and SYBR Green I Stain were purchased from Invitrogen (Carlsbad, CA, USA). Cellular activation of signaling kits for extracellular signal-regulated kinase (ERK), 2-(2-diamino)-3-methoxyphenyl-4H-1-benzopyran-4-one (PD98059), Akt, PI3K, and P38 2-(4-morpholinyl)-8-phenyl-4H-1-benzopyran-4-one (LY294002) was purchased from Cell Signaling Technology (Beverly, MA, USA). ExScript RT reagent kit and SYBR *Premix Ex Taq* (perfect real time) were obtained from TaKaRa (DaLian, China). Rabbit anti-human PAR-1 and rabbit anti-huamn PAR-2 polyclonal antibodies were purchased from Santa Cruz Biotechnology (Santa Cruz, CA, USA). FITC-conjugated mouse anti-human CD4 monoclonal, PE-conjugated mouse anti-human CD8 monoclonal, Percp-cy5.5-conjugated mouse anti-human TNF monoclonal, FITC-conjugated mouse anti-human IFN monoclonal, PE-conjugated mouse anti-human IL-4 monoclonal, APC-conjugated mouse anti-human CD25 monoclonal, and APC-conjugated mouse anti-human IL-17 monoclonal antibodies were purchased from eBioscience. Lymphocyte Isolation Kit I was from Miltenyi Biotec (Bergisch Gladbach, Germany). All other reagents were of analytic grade and obtained from Sigma (St Louis, MO, USA).

### 2.2. Isolation and Culture of T Cells

Human T cells were isolated from peripheral blood mononuclear cells (PBMCs) by a MACS system with T Cell Isolation Kit I according to the manufacturer's protocol. In brief, PBMCs were isolated from fresh blood donated by healthy volunteers, 100 mL from each individual per visit. The informed consent from each volunteer and agreement with the ethical committee of the First Affiliated Hospital of Nanjing Medical University were obtained. After being separated from red blood cells by Ficoll-Paque density gradient, PBMCs were collected and incubated with microbead-linked anti-CD3 monoclonal antibody for 15 min at 8°C. CD3+ T cells were separated from other cells by passing through a magnetic cell separation system. For purity analysis, the cells were resuspended in PBS and incubated with PE-conjugated monoclonal antibody against human CD3 for 1 h. The purity of T cells was consistently more than 95% and cell viability was more than 98%. The purified CD3+ T cells were then used for the further cell challenge tests.

### 2.3. Purified T-Cell Challenge

T cells were cultured in 24-well culture plates at a density of 5 × 10^5^cells/well in RPMI 1640 medium containing 10% NCS at 37°C for 2 h with 5% CO_2_, respectively. The culture supernatants were then removed and cells were washed twice with fresh serum-free RPMI 1640 medium at 300 g for 10 min. For challenge experiments, cells were exposed to various doses of thrombin (0.01–3.0 *μ*g/mL, 1 U = 0.5 *μ*g, 1 U/mL = 5.6 nM, U = NIH unit), trypsin (0.01–0.3 *μ*g/mL, 1 *μ*g/mL = 42 nM), tryptase (0.25–2.0 *μ*g/mL, 1 *μ*g/mL = 7.4 nM), and elastase (0.01–0.3 *μ*g/mL, 1 *μ*g/mL = 34 nM, 1 U/mL = 1700 nM) with or without their inhibitors; and to agonist peptides of PAR-1, PAR-2 and PAR-3 (all at 0.1–100 *μ*M) and their reverse peptides, respectively, for 16 h before the culture, supernatants were harvested and stored at −40°C till use. The cell pellet was used for flow cytometry analysis.

### 2.4. Real-Time PCR Analysis of TNF Gene Expression in Purified T Cells

Quantitative expression of TNF mRNAs in T cells was determined by real-time PCR following the manufacture's protocol. Briefly, after synthesizing cDNA from the total RNA by using ExScriptTM RT reagent kit, real-time PCR was performed by using SYBR Premix Ex Taq on the ABI Prism 7000 Sequence Detection System (Perkin Elmer Applied Systems, Foster City, CA, USA). Each reaction contains 12.5 *μ*L of 2 × SYBR green Master Mix, 1 *μ*L of 10 *μ*M of primers, 1 *μ*L of the cDNA, to a total volume of 25 *μ*L. The thermal cycling conditions included an initial denaturation step at 50°C for 2 min, 95°C for 10 min; 40 cycles at 95°C for 15 s, annealing temperatures at 60°C for 30 s, and extension at 72°C for 30 s.

The sequences of PCR primers for TNF and *β*-actin were 5′-CCCCAGGGACCTCTCTCTAATC-3′ (forward) and 5′-GGTTTGCTACAACATGGGCTACA-3′ (reverse); 5′-AGGGGCCGGACTCGTCATACT-3′ (forward), and 5′-GGCGGCAACACCATGTACCCT-3′ (reverse), respectively.

Consequently, at the end of the PCR cycles, specificities of the amplification products were controlled by dissociation curve analysis. Expression of mRNA in each sample was finally determined after correction with *β*-actin expression. The gene specific threshold cycle (Ct) for each sample (ΔCt) was corrected by subtracting the Ct for the housekeeping gene *β*-actin. Untreated controls were chosen as the reference samples, and the ΔCt for all experimental samples was subtracted by the ΔCt for the control samples (ΔΔCt). The magnitude change of test gene mRNA was expressed as 2 − ΔΔCt. Each measurement of a sample was conducted in duplicate.

### 2.5. Western Blot Analysis of Signal Transduction Pathways in Purified T Cells

T cells were preincubated with 50 *μ*M of PD98059, 20 *μ*M of LY294002, or medium alone for 30 min before adding thrombin 3.0 *μ*g/mL, trypsin 0.3 *μ*g/mL, or medium alone for 30 min, 2 h, or 6 h. The cells were lysed in a buffer containing 20 mM of Tris-HCl (pH 7.4), 137 mM of NaCl, 10% glycerol, 1% Triton X-100, 2 mM of EDTA, 25 mM of *β*-glycerophosphate, 2 mM of sodium pyrophosphate, and 0.5 mM of dithiothreitol at 4°C for 30 min. Cell debris was removed by centrifugation of the lysate at 12,000 ×g for 10 min. The supernatants were mixed with equal volumes of 2x sodium dodecyl sulphate (SDS) sample buffer and heated to 100°C for 10 min. An equal volume of sample was fractionated by SDS-PAGE on a 10% acrylamide gel and transferred onto polyvinylidene difluoride (PVDF) membranes with a Bio-Rad transfer system, according to the manufacturer's instructions. After blocking nonspecific binding sites with 5% BSA in TBST (50 mM of Tris, 0.15 M of NaCl, 0.1% Tween 20, pH 7.6) for 1 h, membranes were probed with phospho-ERK1/2, phospho-Akt, phospho-p38, or phospho-PI3k antibodies at 4°C overnight, followed by incubation with HRP-conjugated secondary antibodies. Immunoreactive bands were visualized by using enhanced chemiluminescence reagents according to the manufacturer's protocol. Densitometry analysis of immunoblots was carried out using Quantity One software (Bio-Rad, USA).

### 2.6. Determination of Cytokines

The levels of TNF in culture supernatants were measured with OptEIA ELISA kits according to the manufacturer's instructions. The plates were read on a plate reader (Molecular Devices, Menlo Park, CA) with the Softmax data analysis program. The minimum detectable concentration of TNF was 2.2 pg/mL.

### 2.7. Flow Cytometry Analysis

To test the PAR1 and 2 expressions after treatment of trypsin and thrombin, isolated T cells were pelleted by centrifugation at 450 g for 10 min after cells were stimulated with thrombin 3.0 *μ*g/mL, trypsin 0.3 *μ*g/mL, or medium alone for 16 h. For PAR1 and PAR2 staining, cells were incubated with rabbit anti-human PAR1 or PAR2 antibodies at 37°C for 1 h. After washing, cells were incubated with PE-conjugated goat anti-rabbit IgG antibody 37°C for 45 min. After washing, cells were analyzed on a fluorescence-activated cell sorting (FACS) arial flow cytometer with CellDevia software (BD Biosciences, USA).

To test the secretion of TNF from subtypes of T cells, isolated T cells were pelleted by centrifugation at 450 g for 10 min and then fixed and permeabilized by using a cell fixation/permeabilization kit (BD Pharmingen). Briefly, thoroughly resuspended cells were added in 100 *μ*L of BD Cytofix/Cytoperm solution and incubated for 20 min at 4°C. Cells were then incubated with fluorescence labeled anti-human CD4, CD8, CD25, TNF, IFN, IL-4, and IL-17 monoclonal antibodies or isotope control, respectively (at a final concentration of 4 *μ*g/mL) at 4°C for 30 min. After washing, cells were analyzed on a fluorescence-activated cell sorting (FACS) Arial flow cytometer with CellDevia software (BD Biosciences, USA).

### 2.8. Statistical Analysis

The results were shown as mean ± SEM. Differences between groups were tested for significance using the Student's *t*-test. *P* < 0.05 was taken as statistically significant. All statistics were performed with SPSS 13.0 for window (SPSS Inc., Chicago, IL, USA).

## 3. Results

### 3.1. Induction of TNF Release from Purified T Cells by Serine Proteinases

**Figure 1:**
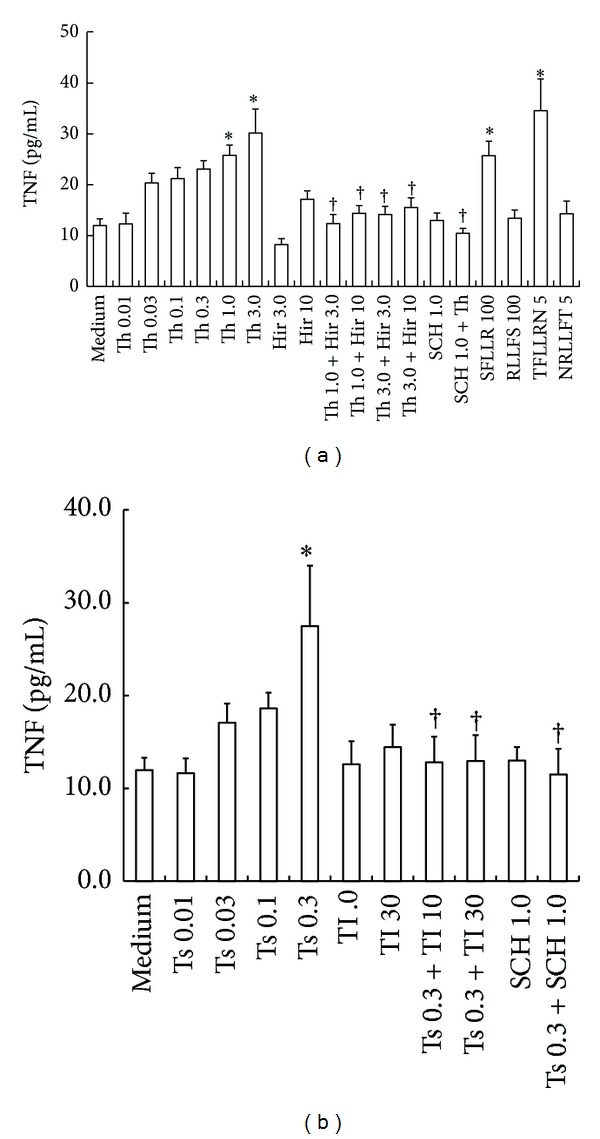
Induction of TNF secretion from human purified T cells by thrombin and trypsin. Cells were incubated with (a) various concentrations of thrombin (Th, *μ*g/mL), SFLLR (*μ*M), TFLLRN (*μ*M), and their reverse peptides RLLFS (*μ*M), NRLLFT (*μ*M), and (b) trypsin (Ts, *μ*g/mL) in the presence or absence of their inhibitors, respectively, for 16 h at 37°C. Values shown are mean ± SEM for four to six independent experiments from different donors. *P < 0.05 compared with the response to medium alone control. ^†^ *P* < 0.05 compared with the response to the corresponding proteinase alone. Hir = hirudin (U/mL), TI = SBTI (*μ*g/mL), and SCH = SCH 79797 (*μ*M).

The purity of T cells was consistently more than 95% (date was shown in Supplementary Material, Figure S1). It has been shown that thrombin, trypsin, and tryptase can induce proinflammatory cytokine IL-6 release from T cells [12], but little is known of serine proteinase-induced TNF release from T cells. Here, we showed that thrombin at concentrations of 1.0 and 3.0 *μ*g/mL provoked TNF release from T cells following 16 h incubation period in a dose-dependent manner. Approximately up to 2.5-fold increase in TNF release was observed when T cells were incubated with thrombin for 16 h. At 6 h following incubation, data (not shown) on both basal and induced TNF release from T cells were inconsistent. This is most likely due to the limitation of the assay sensitivity and relatively low secretion of TNF. PAR-1 agonist peptides, SFLLR-NH_2_ at the concentration of 100 *μ*M and TFLLRN-NH_2_ at the concentration of 5 *μ*M, induced a significant release of TNF at 16 h following incubation. However, RLLFS-NH_2_, a reverse peptide of SFLLR-NH_2_, and NRLLFT-NH_2_, a reverse peptide of TFLLRN-NH_2_, had little effect on release of TNF from T cells (Figure 1(a)).

Hirudin, a specific thrombin inhibitor, was able to inhibit thrombin-induced secretion of TNF. Approximately up to 82.4% inhibition of thrombin-induced secretion of TNF was observed when 3.0 *μ*g/mL of thrombin and 10 U/mL of hirudin were added to T cells for 16 h. Hirudin alone at the concentrations tested had little effect on TNF secretion from T cells. SCH 79797, a PAR-1 antagonist at the concentration of 1 *μ*M, inhibited 89% thrombin-induced TNF release from T cells (Figure 1(a)).

Similarly, trypsin at the concentration of 0.3 *μ*g/mL induced 2.3-fold increase in TNF release from T cells at 16 h (Figure 1(b)). However, tryptase at the concentrations up to 2 *μ*g/mL and elastase at the concentrations up to 6 U/mL had little effect on TNF release from T cells (data not shown). Inhibitors of trypsin, SBTI at the concentrations of 10 and 30 *μ*g/mL, eliminated 0.3 *μ*g/mL trypsin-induced TNF release by a value up to 94.8 and 94.2%, respectively. SBTI alone at the concentrations tested had little effect on TNF secretion from T cells. SCH 79797, a PAR-1 antagonist at the concentration of 1 *μ*M, inhibited 96.8% trypsin-induced TNF release from T cells (Figure 1(b)).

SLIGKV, an agonist peptide of PAR-2 and TFRGAP-NH_2_, an agonist peptide of PAR-3 at the concentrations up to 100 *μ*M, did not appear to have any effect on TNF release from T cells (data not shown).

### 3.2. Real-Time PCR Analysis of Expression of TNF mRNA in Purified T Cells

**Figure 2:**
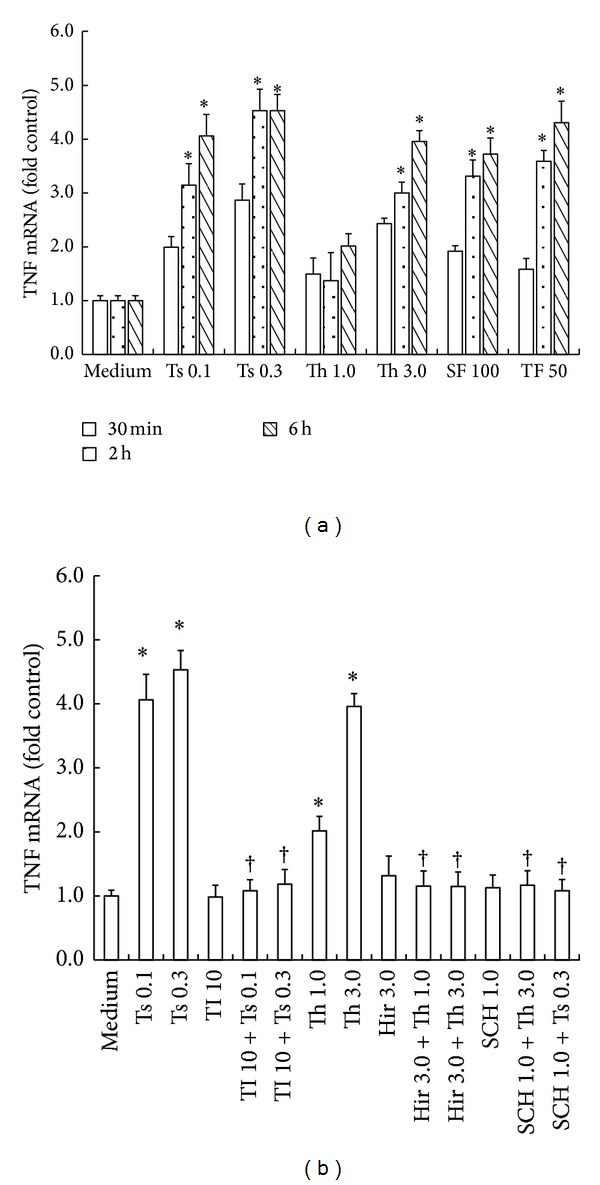
Induction of upregulated expression of TNF mRNA in purified T cells by thrombin and trypsin. (a) Cells were incubated with various concentrations of thrombin (Th, *μ*g/mL), trypsin (Ts, *μ*g/mL), SFLLR-NH_2_ (SF, *μ*M), and TFLLRN-NH_2_ (TF, *μ*M) for 30 min, 2 h or 6 h at 37°C. (b) Cells were incubated with Th (*μ*g/mL) and Ts (*μ*g/mL) in the presence or absence of their inhibitors, respectively for 6 h at 37°C. Values shown are mean ± SEM for four to six independent experiments from different donors. *P < 0.05 compared with the response to medium alone control. ^†^ *P* < 0.05 compared with the response to the corresponding proteinase alone. Hir = hirudin (U/mL), TI = SBTI (*μ*g/mL), and SCH = SCH 79797 (*μ*M).

In order to confirm the findings above, we investigated the influence of the serine proteinases on the expression of TNF mRNA in T cells. It was found that the expression of TNF mRNA was upregulated when T cells were incubated with thrombin at 1 and 3 *μ*g/mL for 2 and 6 h. The maximum enhanced expression of TNF mRNA was 4.2-fold over baseline control (Figure 2(a)) after 6 h incubation. Hirudin, a specific thrombin inhibitor at the concentration of 3 U/mL, completely abolished thrombin-induced upregulated expression of TNF mRNA after 6 h incubation (Figure 2(b)).

Trypsin at the concentration of 0.3 *μ*g/mL also induced increased expression of TNF mRNA by a value up to approximately 4.0-fold in T cells (Figure 2(a)), which was completely blocked by SBTI (Figure 2(b)). Similarly, SCH 79797 at the concentration of 1 *μ*M inhibited both thrombin- and trypsin-induced upregulated expression of TNF mRNA in T cells by a value up to 72 and 72.5%, respectively (Figure 2(b)).

SFLLR-NH_2_ at the concentration of 100 *μ*M and TFLLRN-NH_2_ at the concentration of 5 *μ*M significantly increase the expression of TNF mRNA at 2 and 6 h following incubation (Figure 2(a)). But RLLFS-NH_2_, a reverse peptide of SFLLR-NH_2_, and NRLLFT-NH_2_, a reverse peptide of TFLLRN-NH_2_, had little effect on expression of TNF mRNA in T cells (data not shown).

At the same time, neither thrombin nor trypsin showed obvious effect on the expression of PAR-1 and PAR-2 (data not shown).

### 3.3. Upregulated Expression of TNF in Subtypes of T Cells

**Figure 3:**
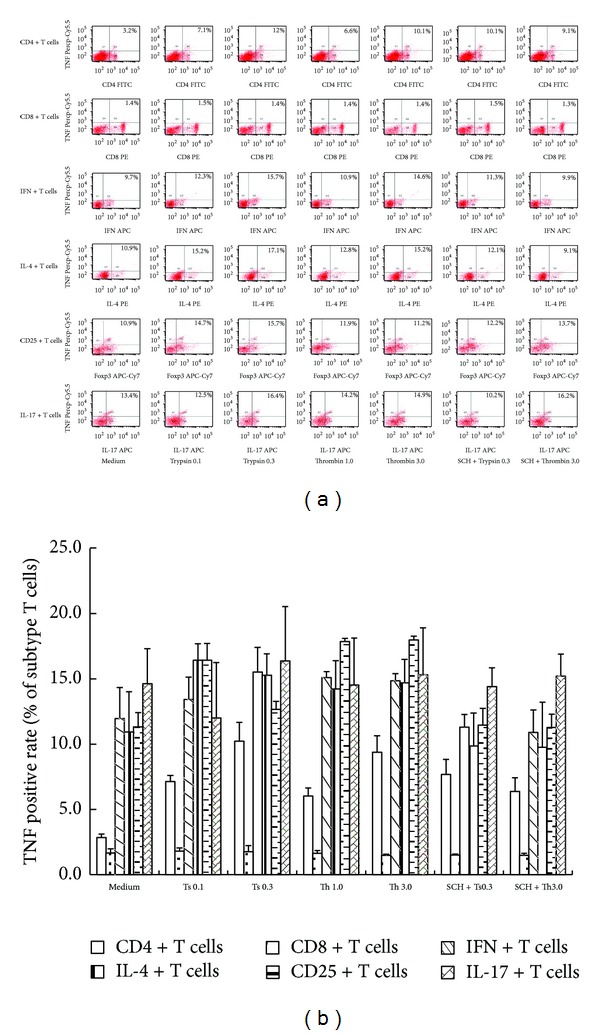
Induction of upregulated expression of TNF in subtypes of purified T cells. Isolated T cells were incubated with trypsin or thrombin for 16 h at 37°C before being analyzed by flow cytometer. (a) Numbers within the large gated regions indicate the percentage of TNF expression cells among different subtypes of T cells. (b) The mean ± SEM data represented the percentage of TNF + cells in different subtypes of T cells indicated for four separate experiments. *P < 0.05 in comparison with medium alone control. ^†^ *P* < 0.05 compared with the response to the corresponding uninhibited control.

It is wellknown that there are numerous subtypes of T cells and each of them has distinctive functions. We, therefore, investigated subtypes of T cells by flow cytometer analysis in order to determine the subtypes that upregulate TNF in response to trypsin or thrombin. The results showed that trypsin and thrombin induced upregulated expression of TNF in CD4+ T cells, but not CD8+ T cells, following 16 h incubation period. Among CD4+ T cells, trypsin and thrombin enhanced TNF expression in IL-4+ or CD25+ T cells, but not in IFN+ or IL-17+ T cells. SCH 79797 was able to inhibit enhanced TNF expression induced by trypsin and thrombin (Figures 3(a) and 3(b)).

### 3.4. Effect of PD98059 and LY294002 on Release and Gene Expression of TNF

**Figure 4:**
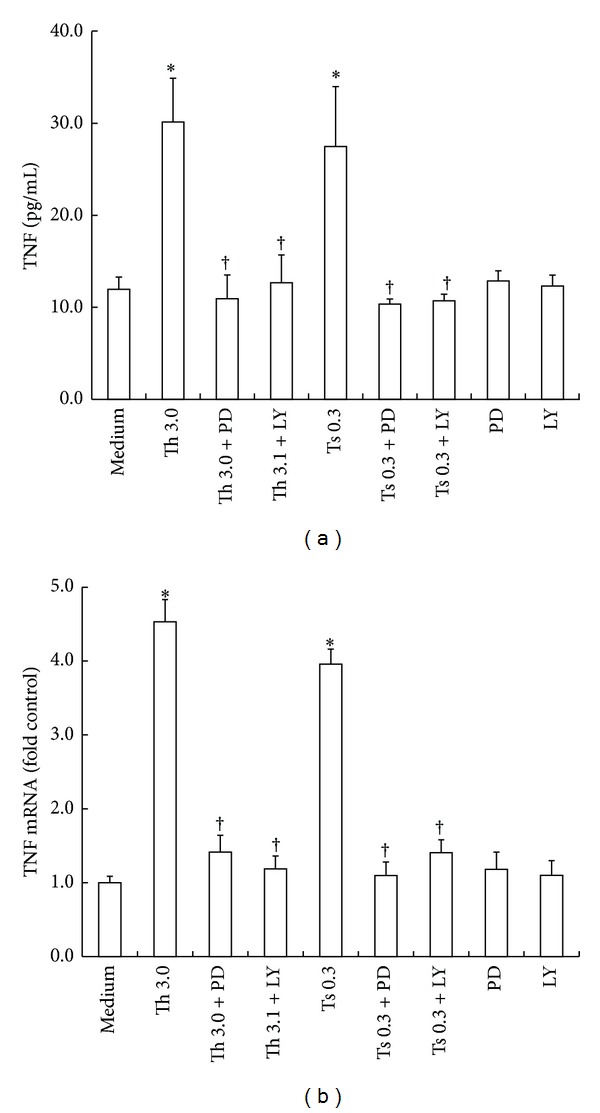
Effect of PD98059 and LY294002 on release and gene expression of TNF in purified T cells. PD98059 (PD, 50 *μ*M) or LY294002 (LY, 20 *μ*M) was preincubated with T cells at 37°C for 30 min before 3.0 *μ*g/mL of thrombin and 0.3 *μ*g/mL of trypsin being added for 6 or 16 h. (a) Cells were collected for analysis of TNF release from T cells at 16 h following incubation. (b) Cells were collected for analysis of gene expression of TNF in T cells at 6 h following incubation. Values shown are mean ± SEM for four to six independent experiments from different donors. *P < 0.05 compared with the response to medium alone control. ^†^ *P* < 0.05 compared with the response to the corresponding proteinase alone. Th = thrombin (*μ*g/mL), Ts = trypsin (*μ*g/mL), LY = LY294002 (*μ*M), and PD = PD98059 (*μ*M).

In order to examine signal transduction pathways of thrombin and trypsin, T cells were preincubated with PD98059, LY294002, or medium alone for 30 min before adding thrombin 3.0 *μ*g/mL, trypsin 0.3 *μ*g/mL, or medium alone for 16 h. Following 16 h incubation period, PD98059 an inhibitor of MAPK pathway, and LY294002, an inhibitor of PI3K, completely blocked thrombin- and trypsin-induced release of TNF (Figure 4(a)).

Furthermore, PD98059 inhibited thrombin- and trypsin-induced upregulation of expression of TNF mRNA by a value up to 91.2 and 98.6%, and LY294002 eliminated thrombin- and trypsin-induced expression of TNF mRNA by 95.5 and 83.2% in T cells following 6 h incubation (Figure 4(b)).

### 3.5. Effect of PD98059 on Phosphorylation of ERK in Purified T Cells

**Figure 5:**
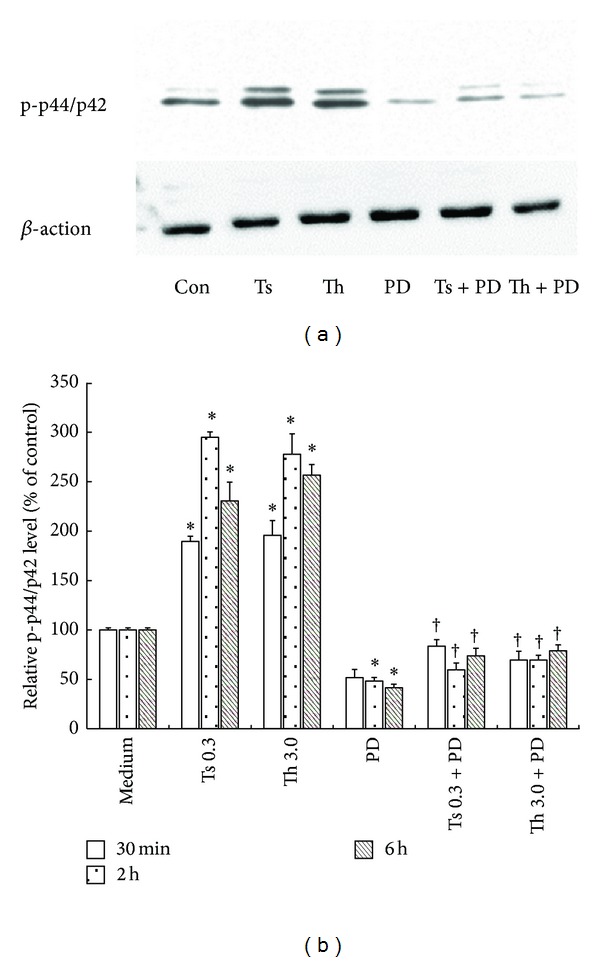
Western blot analysis of influence of PD98059 on thrombin- and trypsin-induced phosphorylation of ERK in purified T cells. PD98059 (PD, 50 *μ*M) was preincubated with T cells at 37°C for 30 min before 3.0 *μ*g/mL of thrombin and 0.3 *μ*g/mL of trypsin being added for 30 min and 2 and 6 h. (a) Cells were treated with thrombin and trypsin for 2 h. (b) The relative levels of phospho-ERK1/2 were expressed as the ratio to *β*-actin, an internal control (house keeping protein). The values shown are mean ± SD for four separate experiments. *P < 0.05 compared with the response to medium alone. ^†^ *P* < 0.05 compared with the response to the corresponding proteinase alone.

Thrombin (3 *μ*g/mL) and trypsin-(0. 3 *μ*g/mL) induced enhanced phosphorylation of ERK1/2 in T cells following 0.5, 2, and 6 h incubation periods. However, thrombin and trypsin did not significantly affect phosphorylation of P38 in T cells following 0.5, 2, and 6 h incubation periods (Date was shown in Supplementary Material, Figure S2). PD98059 was able to completely block thrombin- and trypsin-induced phosphorylation of ERK1/2 when it was preincubated with T cells for 30 min. PD98059 also inhibited basal phosphorylation of ERK1/2 in T cells (Figure 5).

### 3.6. Effect of LY294002 on Akt Phosphorylation in Purified T Cells

**Figure 6:**
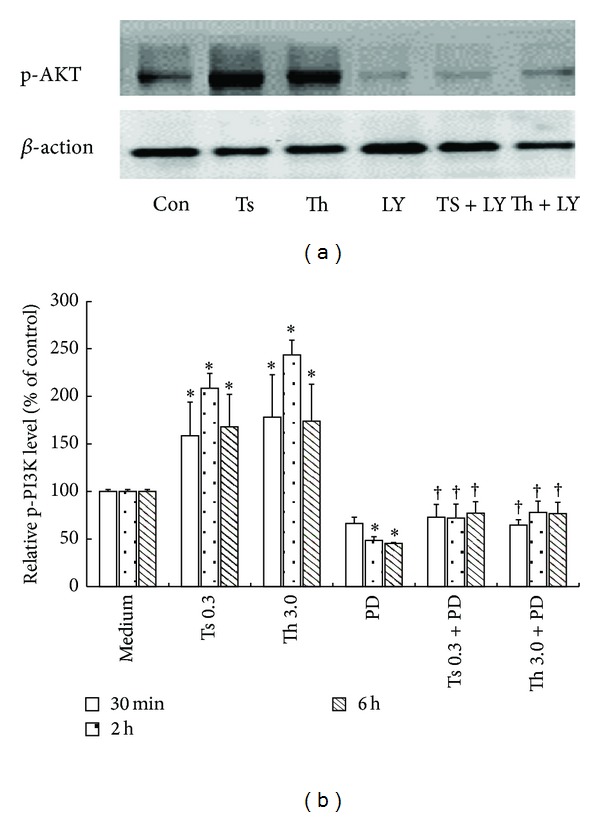
Western blot analysis of influence of LY294002 on thrombin- and trypsin-induced phosphorylation of AKT in purified T cells. LY294002 (LY, 20 *μ*M) was preincubated with T cells at 37°C for 30 min before 3.0 *μ*g/mL of thrombin and 0.3 *μ*g/mL of trypsin being added for 0.5, 2, and 6 h. (a) Cells were treated with thrombin and trypsin for 2 h. (b) The relative levels of phospho-Akt were expressed as the ratio to *β*-actin, an internal control (housekeeping protein). The values shown are mean ± SD for four separate experiments. *P < 0.05 compared with the response to medium alone. ^†^ *P* < 0.05 compared with the response to the corresponding proteinase alone.

Thrombin at a concentration of 3 *μ*g/mL and trypsin at a concentration of 0.3 *μ*g/mL induced significantly increased phosphorylation of Akt in T cells following 0.5, 2, and 6 h incubation periods. However, thrombin and trypsin did not significantly affect phosphorylation of PI3k in T cells following 0.5, 2, and 6 h incubation periods (date was shown in Supplementary Material, Figure S3). LY294002 was able to block thrombin- and trypsin-induced phosphorylation of Akt when it was incubated with T cells for 30 min. LY294002 also diminished basal phosphorylation of Akt in T cells (Figure 6).

## 4. Discussion

We discovered in the present study that serine proteinases thrombin and trypsin, but not tryptase induced TNF release from human T cells. Since TNF is a potent proinflammatory cytokine, our observation is likely to add some novel information for, understanding of actions of serine proteinases in causing inflammation.

As little as 1.0 *μ*g/mL of thrombin was able to induce significant TNF release from T cells, suggesting this proteinase is a potent secretagogue of TNF. This concentration of thrombin should be easily achieved in blood, particularly when the processes of platelet aggregation and coagulation are initiated [21]. Inhibition of thrombin-induced TNF release by a specific inhibitor of thrombin and hirudin indicates that action of thrombin on T cells was dependent on the enzymatic activity of this serine proteinase. There are 3 receptors for thrombin on cells, including PAR-1, PAR-3, and PAR-4 [2, 3]. Since PAR-1 agonist peptides SFLLR-NH2, and TFLLRN-NH2, but not PAR-3 agonist peptide TFRGAP-NH2 were capable of stimulating TNF release, a PAR-1 antagonist SCH 79797 [22] almost completely abolished thrombin-induced TNF release from T cells, and purified human T cells do not express PAR-4; the action of thrombin on T cells is most likely through activation of PAR-1. Our previous report which found thrombin-induced IL-6 secretion from human peripheral blood T cells may support our current findings [16].

While little information is available on induction of TNF release from T cells by trypsin, the ability of trypsin to stimulate IL-6 secretion from T cells [16] may support the anticipation that trypsin is capable of inducing cytokine release from T cells. As little as 0.3 *μ*g/mL of trypsin was able to provoke TNF secretion from T cells proved that it is a potent stimulus of TNF release. As for thrombin, inhibitor of trypsin SBTI was able to inhibit trypsin-induced TNF release from T cells, indicating that an intact catalytic site is required for the serine proteinase to stimulate TNF release. Since PAR-1 is one of three receptors of trypsin, PAR-1 agonist peptides SFLLR-NH_2_ and TFLLRN-NH_2_ are capable of stimulating TNF release from T cells, and SCH 79797 almost completely abolished trypsin-induced TNF release from T cells, the action of trypsin on T cells is most likely through activation of PAR-1. PAR-2 is also a receptor of trypsin. Since PAR-2 agonist peptide SLIGKV-NH_2_ and tryptase are not capable of stimulating TNF release from T cells, the action of trypsin on T cells is not likely through activation of PAR-2.

Trypsin- and thrombin-induced upregulated expression of TNF was observed in CD4+, IL-4+ or CD25+ T cells, indicating that IL-4+, and CD25+ T cells are major sources of TNF. While little information on the relationship between CD25+ T cells and TNF is available, a study which found that the percentage of CD4(+)CD25(+) T cells were significantly high, but the percentage of FoxP3(+) cells were low in allergic rhinitis patients, and that IL-4, IL-5, and TNF levels in nasal lavage fluids were high indicates that the increased TNF release may be from CD4(+)CD25(+), nonregulatory T cells [23]. We believe that the current study is the first work that demonstrates coexpression of CD25 and TNF in the subtype of CD4(+) T cells. Similarly, we clearly found that IL-4+ T cells express enhanced TNF, though little information on co-expression of IL-4 and TNF in T cells is available. This finding implicates that trypsin and thrombin may be involved in the inflammation through induction of TNF release from IL-4+ or CD25+ T cells. It was demonstrated that nickel-specific CD4+ T cell lines [24] and Th17 cells [25] corelease IL-17 and TNF, but trypsin- and thrombin-induced TNF release appears not from IL-17+ T cells as TNF expression in IL-17+ T cells was not upregulated by these two proteinases.

MAPK/ERK pathway is the signaling pathway that is most likely involved in the thrombin- and trypsin-induced TNF release from highly purified T cells, as PD98059, an inhibitor of MAPK/ERK pathway, almost completely blocked thrombin- and trypsin-provoked phophorylation of ERK and TNF release. While little information on signaling pathways associated with PAR-1 signaling in purified T cells is available, the previous reports that PAR-1 agonists activated MAPK/ERK and p38 MAPK signaling pathways in dermal [26] and cardiac fibroblasts [27] may support our current observation that MAPK/ERK pathway is the signaling pathway that is most likely involved in the thrombin- and trypsin-induced TNF release. In addition, PI3K/Akt signaling pathway seems also to be involved in thrombin and trypsin induced TNF secretion, as LY294002 an inhibitor of PI3K/Akt signaling pathway partially diminished thrombin and trypsin induced TNF secretion and completely abolished thrombin and trypsin provoked phosphorylation of Akt. This finding is in the same line with the report, which showed that thrombin stimulated enhance PI3K activity in hamster embryonic fibroblasts [28], but different from our previous report, which showed that thrombin did not enhanced PI3K activity in human dermal fibroblasts [29]. The discrepancy between these studies may be due to the difference in cell origin and species.

TNF is a member of a growing family of peptide mediators comprising at least 19 cytokines, including lymphotoxin-*α*, Fas ligand, and CD40 ligand. The family is now considered as central mediators of a broad range of biological activities in protective immune responses against a variety of infectious pathogens. On the other hand, TNF also exerts host-damaging effects in sepsis and autoimmune disease [30, 31]. These findings indicate that TNF is one of key mediators of inflammation; therefore, our current study is of importance in understanding TNF-related inflammation and the mechanism of proteinase-induced cytokine production in T cells.

## 5. Conclusions

In conclusion, it is discovered in the present study that serine proteinases thrombin and trypsin are potent stimuli of TNF secretion from highly purified T cells. Their actions on T cells depend on their enzymatic activities and are likely through activation of PAR-1. Stimulation of TNF secretion from T cells by serine proteinases further proved that these proteinases are actively involved in the pathogenesis of inflammation and regulation of immune response in man.
